# Federation of Infectious Diseases Societies of Southern Africa guideline: Recommendations for the detection, management and prevention of healthcare-associated *Candida auris* colonisation and disease in South Africa

**DOI:** 10.4102/sajid.v34i1.163

**Published:** 2019-09-26

**Authors:** Nelesh P. Govender, Theunis Avenant, Adrian Brink, Vindana Chibabhai, Joy Cleghorn, Briette du Toit, Chetna Govind, Elsie Lewis, Warren Lowman, Hleziphi Mahlangu, Caroline Maslo, Angeliki Messina, Mervyn Mer, Kim Pieton, Sharona Seetharam, Charlotte Sriruttan, Karin Swart, Erika van Schalkwyk

**Affiliations:** 1 National Institute for Communicable Diseases, Centre for Healthcare-Associated Infections, Antimicrobial Resistance and Mycoses, Division of the National Health Laboratory Service, Johannesburg, South Africa; 2 Faculty of Health Sciences, University of the Witwatersrand, Johannesburg, South Africa; 3 Faculty of Health Sciences, University of Cape Town, Cape Town, South Africa; 4 Kalafong Provincial Tertiary Hospital and Faculty of Health Sciences, University of Pretoria, Pretoria, South Africa; 5 Ampath Laboratories, Cape Town, South Africa; 6 National Health Laboratory Service, Johannesburg, South Africa; 7 Charlotte Maxeke Johannesburg Academic Hospital, Johannesburg, South Africa; 8 Life Healthcare Group, Johannesburg, South Africa; 9 Mediclinic Southern Africa, Cape Town, South Africa; 10 Lancet Laboratories, Durban, South Africa; 11 Steve Biko Pretoria Academic Hospital, Pretoria, South Africa; 12 WITS Donald Gordon Medical Centre and Vermaak and Partners Pathologists, Johannesburg, South Africa; 13 Clinix Health Group, Johannesburg, South Africa; 14 Netcare Hospitals Limited, Johannesburg, South Africa; 15 Chris Hani Baragwanath Academic Hospital, Johannesburg, South Africa; 16 Lancet Laboratories, Cape Town, South Africa; 17 National Institute for Communicable Diseases [Centre for Healthcare-Associated Infections, Antimicrobial Resistance and Mycoses], a Division of the National Health Laboratory Service, Johannesburg, South Africa

**Keywords:** *Candida auris*, Candidaemia, Infection Control, Antifungal Treatment, Diagnosis, Antifungal Stewardship

## Abstract

*Candida auris* has been detected at almost 100 South African hospitals, causing large outbreaks in some facilities, and this pathogen now accounts for approximately 1 in 10 cases of candidaemia. The objective of this guideline is to provide updated, evidence-informed recommendations outlining a best-practice approach to prevent, diagnose and manage *C. auris* disease in public- and private-sector healthcare settings in South Africa. The 18 practical recommendations cover five focus areas: laboratory identification and antifungal susceptibility testing, surveillance and outbreak response, infection prevention and control, clinical management and antifungal stewardship.

## Introduction

Cases of *Candida auris* were first reported from East Asia in 2009, although earlier cases have since been detected in culture repositories from as early as 1996.^1,2,3^ By 2018, cases of *C. auris* had been reported from all six inhabited continents.^3,4^ Of particular concern is that large outbreaks of *C. auris* have been reported from resource-limited settings in Asia, Africa and South and Central America.^5,6,7,8^ For instance, *C. auris* has been detected in almost 100 South African hospitals, causing large outbreaks at some facilities, and this pathogen now accounts for approximately 1 in 10 cases of candidaemia.^7,9^

The reasons for the dramatic emergence of *C. auris* as a pathogen in healthcare settings are not clear. We know that East Asia, South Asia, Africa and South America have unique *C. auris* clades separated from other clades by tens of thousands of single nucleotide polymorphisms.^10^ This is consistent with the hypothesis that *C. auris* emerged independently and simultaneously on several continents. While *C. auris* is likely to have an environmental reservoir outside the healthcare setting, this has yet to be established. Several intrinsic properties of the pathogen probably facilitated its rapid spread in hospitals. *C. auris* produces biofilms.^11,12,13^ While this fungus rarely colonises the hands of healthcare workers, it can survive for prolonged periods in the immediate environment around infected or colonised patients, and in a recent outbreak investigation, it was found to contaminate re-useable patient equipment.^13,14,15^ *C. auris* is also relatively resistant to some chemical disinfectants.^16,17^ Transmission can thus occur from an infected or colonised person, the patient care environment or re-useable equipment to a susceptible person. In South Africa, *C. auris* has become a common healthcare-associated pathogen in the same geographic region where azole-resistant *Candida parapsilosis* was first described.^18^ It is likely that inadequate antifungal stewardship (AFS) and infection prevention and control (IPC) programmes are the underlying drivers of the emergence and transmission of these azole-resistant pathogens. Infection prevention and control and antifungal stewardship are two key areas covered in this guideline document. *C. auris* causes healthcare-associated outbreaks and is a public health concern; therefore, locally relevant recommendations for appropriate surveillance and outbreak response activities are essential and covered in this article.

Without a clear laboratory algorithm, *C. auris* is often misidentified by routine methods.^19^ Misidentification delays initiation of appropriate antifungal treatment and rapid institution of IPC measures. *C. auris* causes a wide range of invasive and non-invasive infections and colonises various body sites. Identification of species level is not routine for isolates from non-sterile sites; therefore, *C. auris* would be missed unless this is specifically looked for.^20^ *C. auris* is almost universally resistant to fluconazole and has variable susceptibility to other classes of antifungals.^5,10,21^ The lack of clinically relevant breakpoints currently limits interpretation of minimum inhibitory concentrations (MICs) and hence guidance for individual patient treatment.^22^ This guideline includes recommendations for identifying and performing antifungal susceptibility testing for *C. auris*.

Owing to its relatively recent emergence, patients with *C. auris* infection were not included in pre-registration clinical trials for currently available antifungal agents. Recommendations for antifungal treatment of *C. auris* disease are thus extrapolated from evidence for *Candida* infections with other species and there are no published recommendations for low- and middle-income countries.^23^ Based on South African surveillance data, the following independent risk factors have been identified for *C. auris* candidaemia: prior antifungal treatment, older patients, prolonged hospitalisation, admission to private-sector facilities and having a central venous catheter *in situ*.^9^ These risk factors are not sufficiently specific and so healthcare workers need to maintain a high index of suspicion for *C. auris,* particularly in settings where this pathogen is endemic.

The objective of this guideline is to provide updated, evidence-informed recommendations outlining a best-practice approach to prevent, diagnose and manage *C. auris* disease in public- and private-sector healthcare settings in South Africa. The recommendations contained in this guideline are not all specific to *C. auris* and some sections (e.g. IPC, AFS and antifungal treatment) may be applied to healthcare-associated infections caused by other *Candida* species. This guideline is aimed at medical practitioners, nurses, IPC practitioners, clinical pharmacists, clinical microbiologists, laboratory technical personnel and members of interdisciplinary IPC and/or antimicrobial stewardship hospital committees who are involved in the diagnosis, prevention or management of *C. auris* in a healthcare setting. Although these recommendations were designed for acute-care settings, aspects of this guideline may also be applicable to chronic-care settings. Implementation of the recommendations should be informed by local context, including epidemiology of fungal infections and prevalence of other comorbidities, availability of resources, the organisation and capacity of the healthcare system and anticipated cost-effectiveness of the recommendations.

## Methods

Previously, no South African guideline on candidiasis has been published. For this guideline, the Federation of Infectious Diseases Societies of Southern Africa (FIDSSA) convened a multidisciplinary panel. Nominations to the guideline development group were requested from the chairpersons of the following professional societies or groups: South African Society for Clinical Microbiology (including National Health Laboratory Service and private pathology practices), South African Paediatric Infectious Diseases Society, Infectious Diseases Society of Southern Africa, Infection Control Society of South Africa (including public- and private-sector IPC practitioners), South African Antibiotic Stewardship Programme and Critical Care Society of Southern Africa. In addition, members were nominated from the following institutions or private healthcare groups: National Institute for Communicable Diseases (NICD), Life Healthcare Group, Netcare, Clinix and Mediclinic Southern Africa.

An in-person meeting was convened in Johannesburg on 06 July 2017 to discuss and propose recommendations. The 19-member panel comprised seven clinical microbiologists, one paediatric infectious diseases (ID) specialist, one adult ID specialist, one critical care physician, five IPC nurse practitioners, one general medical practitioner, two medical epidemiologists and one clinical pharmacist. The proceedings of the meeting were recorded and transcribed. At this meeting, members were assigned to writing groups for each section. The writing groups subsequently met in person or via teleconference or corresponded by email to draft each set of recommendations. Compiled draft recommendations were presented by N.P.G. for discussion on 04 November 2017 at the 7th FIDSSA conference in Cape Town. The guideline development group then re-convened by teleconference on 27 November 2017.

Owing to the paucity of high-quality evidence specifically relevant to *C. auris*, systematic reviews were not conducted for each focus area prior to developing this guideline. The chairperson (N.P.G.) conducted a literature review prior to the July 2017 meeting and uploaded all relevant full-text articles or documents to a cloud-based file share service. Each writing group also conducted separate reviews of the literature. The quality of evidence was not specifically rated for each recommendation. The strength of each recommendation was also not quantified. These recommendations should thus be considered to be based on expert opinion. The guideline document was circulated to an external peer review group in May 2018. This group included five nominees from the professional societies listed above who had not been involved in developing the guideline (Sean Wasserman, Jeremy Nel, Colleen Bamford, Shaheen Mehtar and Lesley Devenish). The guideline was endorsed by the FIDSSA, South African Society for Clinical Microbiology, South African Paediatric Infectious Diseases Society, Infectious Diseases Society of Southern Africa, Infection Control Society of South Africa and the Critical Care Society of Southern Africa.

### Section 1: Laboratory identification and antifungal susceptibility testing

#### Recommendation 1.1: When should the diagnostic laboratory suspect *Candida auris*?

**FIGURE 1:**
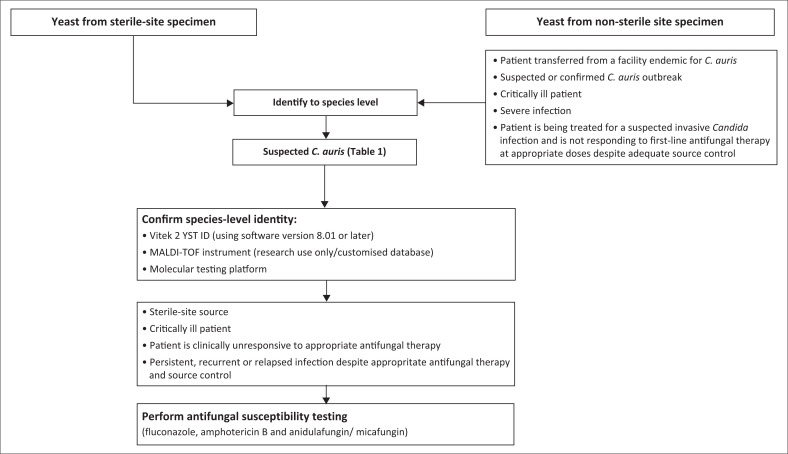
Laboratory testing algorithm for identification of *Candia auris*.

Current commercial automated or biochemical identification systems misidentify *C. auris*, often in a predictable manner. Yeasts identified as any of the organisms by the corresponding presumptive identification method (see Table 1) should be suspected to be *C. auris*, particularly if found to be fluconazole resistant, and tested further as per the recommended laboratory algorithm (see Figure 1).

**TABLE 1:** When to suspect *Candia auris* in the clinical laboratory.^19^

Instrument/biochemical kit	Identification obtained	What to do next?
API 20C AUX or ID32C	*Rhodotorula glutinis*	If colonies are not pink or yeast is urease-negative, refer†
Auxacolor	*Saccharomyces*	Consider *C. auris* and refer†
Microscan	*Candida famata*	Consider *C. auris* and refer†
Microscan	*Candida lusitaniae, Candida guilliermondii, Candida parapsilosis, Candida catenulata*	Not possible to detect *C. auris* unless the yeast ID is confirmed with another method and/or fluconazole resistance is documented
Vitek 2 YST	*Candida haemulonii* if software update is not loaded	If fluconazole resistant, treat as *C. auris* and refer†
Vitek 2 YST	*Candida auris* if software version 8.01 is loaded	Report as *Candida auris*
Vitek MS MALDI	*Candida auris* if research use only (RUO) library is used	Report as *Candida auris*
Bruker BioTyper MALDI	*Candida auris* if full/partial extraction method and RUO library is used	Report as *Candida auris*

*Source*: Mizusawa M, Miller H, Green R, et al. Can multidrug-resistant *Candida auris* be reliably identified in clinical microbiology laboratories? J Clin Microbiol. 2017;55(2):638–640. https://doi.org/10.1128/JCM.02202-16

† , Refer to a laboratory with Vitek 2 YST software version 8.01 or MALDI-TOF or molecular testing platform.

Early identification of *C. auris* is important to guide appropriate antifungal treatment and to implement appropriate IPC measures. The laboratory should suspect *C. auris* when specimens are submitted from facilities or units known to be endemic for this pathogen. In a recent South African study, the risk of *C. auris* candidaemia (vs. fungaemia caused by any other *Candida* species) was threefold higher among patients admitted to private-sector hospitals. Other risk factors included prior antifungal treatment, older age, longer hospitalisation before first positive culture and a central venous catheter *in situ*.^9^ Current commercial identification systems often misidentify *C. auris* as the organisms listed in Table 1.^19,20^ *C. auris* is almost uniformly resistant to fluconazole^10^; if a yeast is found to be resistant to fluconazole and the first-line automated or biochemical identification system also yields an unexpected identity (Table 1), consider *C. auris* and refer to a laboratory with Vitek 2 YST software version 8.01 or a matrix assisted laser desorption ionisation-time of flight (MALDI-TOF) mass spectrometry instrument or molecular testing platform.

#### Recommendation 1.2: How should *Candida auris* be identified in the laboratory?

1. Perform species-level identification for all *Candida* isolates cultured from sterile body sites. Ideally, species-level identification should also be obtained for *Candida* isolates cultured from all non-sterile sites. However, in situations where this is not routinely possible, we recommend speciation from non-sterile sites:
  - if a patient is transferred from a facility known to be endemic for *C. auris*
  - during suspected or confirmed *C. auris* outbreaks
  - among critically ill patients
  - for severe infections
  - when a patient is being treated for a suspected invasive *Candida* infection and is not responding to first-line antifungal therapy at appropriate doses despite adequate source control
2. Confirm identification of *C. auris* on a MALDI-TOF instrument, the Vitek 2 YST ID system or by sequencing the multi-copy fungal ribosomal gene (internal transcribed spacer [ITS] or D1/D2 regions).

*Candida auris* isolates are frequently misidentified in the clinical laboratory. They are germ tube-negative yeasts and are able to grow at relatively high temperatures (42°C).^11^ They appear pink or purple on chromogenic *Candida* agar (CHROMagar, Paris, France). Confirmation of species-level identification can be performed using either a MALDI-TOF instrument (such as VITEK MS [Biomérieux, Marcy l’Étoile, France] or Bruker Biotyper [Bruker, Billerica, MA, USA] using the corresponding research use only or customised databases) or the Vitek 2 YST ID system (Biomérieux) updated with software version 8.01.^19,24^ Molecular identification is the reference standard method.^25,26^ *Candida* should be routinely identified to species level if isolated from a sterile site such as blood, cerebrospinal fluid, tissue, pus from deep abscesses, etc. Not all diagnostic laboratories routinely identify *Candida* species other than *Candida albicans* from non-sterile sites to species level. This may result in under-reporting during outbreaks. The guideline development group believe that species-level identification is particularly important to detect *C. auris* from all specimens for the following reasons: *C. auris* outbreaks may be prolonged and difficult to control; patients who are colonised represent an important reservoir for transmission. *C. auris* is potentially multidrug-resistant, with consistently high fluconazole MICs and occasionally high amphotericin B and echinocandin MICs. Reported cases of therapeutic failure have been documented with azoles and amphotericin B.^3,16,17^

#### Recommendation 1.3: When should antifungal susceptibility testing for *Candia auris* be performed and how should results be interpreted?

1. Perform routine antifungal susceptibility testing if *C. auris* is isolated:
  - from blood or any other sterile site specimen
  - among all critically ill patients
  - from a non-sterile site if the patient is clinically unresponsive to appropriate antifungal therapy
  - if there is persistent, recurrent or relapsed infection despite appropriate antifungal therapy and source control
2. If possible, perform antifungal susceptibility testing using a standardised broth microdilution (BMD) method, Sensititre YeastOne or Etest. Confirm all Vitek 2 amphotericin B MICs by another method.
3. The following agents are recommended for antifungal susceptibility testing: fluconazole (also useful for identification), amphotericin B and anidulafungin/micafungin. Caspofungin MIC testing should be avoided to predict echinocandin resistance.
4. For each antifungal agent that is tested, laboratories should report an MIC.
5. Epidemiologic cut-off (ECOFF) values can be used to categorise isolates as wild type or non-wild type (i.e. mutants) for each antifungal agent. If the MIC ≥ ECOFF for that agent, report to the clinician using a standard clearly worded comment.
6. Laboratories may consider use of cut-off values proposed by the US Centers for Disease Control and Prevention (US CDC)^27^ but should be clear that these are not validated clinical breakpoints, and if the MIC is higher than the proposed cut-off value, provide a report to the clinician using a clearly worded comment, including a recommendation that a clinical microbiologist or ID physician be consulted.
7. Refer all strains with elevated amphotericin B (≥ 2 µg/mL) or anidulafungin/micafungin MICs (≥ 4 µg/mL) for testing at a reference laboratory.

If carefully standardised and quality-controlled, antifungal susceptibility testing can yield reproducible MICs that facilitate selection of the optimal antifungal agent for use in a particular clinical scenario. Most laboratories perform routine testing on isolates from sterile sites. In certain circumstances, outlined in the recommendation above, antifungal susceptibility testing should be performed on non-sterile site isolates. Although very important, an MIC is not the only factor to be considered when selecting an antifungal agent. The ability of an antifungal agent to kill the pathogen may be important for early treatment success and to reduce the chance of persistent, recurrent or relapsed infection.^28^ Some infected body compartments or sites (e.g. the central nervous system, urinary tract, eye and intra-abdominal abscesses) are not easily penetrated by echinocandins and the pharmacokinetics/pharmacodynamics of various agents should be compared.

A standardised reference BMD test is the recommended antifungal susceptibility testing method to resolve discrepancies and to confirm unusual phenotypes. A direct comparison of the European Committee on Antifungal Susceptibility Testing (EUCAST) and US Clinical and Laboratory Standards Institute (CLSI) BMD methods for a *C. auris* isolate collection yielded similar MICs for fluconazole, itraconazole, voriconazole, isavuconazole, posaconazole, anidulafungin, micafungin and amphotericin B.^22^ When CLSI-BMD and the commercial automated Vitek AST-YS07 were compared, there was 100% agreement of MIC_50_ values for voriconazole, caspofungin and micafungin and agreement for fluconazole and flucytosine within two dilutions. Of concern is that Vitek AST-YS07 yielded falsely elevated MICs (MIC_50_ of 8 µg/mL) for amphotericin B compared to the CLSI-BMD MIC_50_ of 1 µg/mL and an Etest MIC_50_ of 0.5 µg/mL.^29^ The guideline development group therefore recommends that all amphotericin B MIC results obtained with Vitek 2 AST-YS07 system should be confirmed with another method. There are no data comparing Sensititre YeastOne or Etest MICs to reference BMD MICs for *C. auris*; however, these methods provide MICs with close approximation to the reference methods for other *Candida* species. Laboratories should avoid testing or reporting caspofungin MICs for detection of echinocandin resistance because this method is subject to error^21^; however, any echinocandin (including caspofungin) can be used for clinical treatment if the pathogen is shown to be echinocandin susceptible. Mutations in the hotspot regions of the FKS genes are usually associated with echinocandin resistance in *C. auris*, although very few laboratories currently perform FKS gene sequencing.

There are currently no clinical breakpoints for *C. auris* and any antifungal agent. As limited clinical and pharmacokinetic/pharmacodynamic data currently preclude the development of such breakpoints, ECOFFs may be helpful. Epidemiologic cut-offs distinguish organisms with and without phenotypically expressed resistance mechanisms for a species and an antifungal agent in a defined test system; within a species, this is the highest MIC of organisms lacking phenotypically expressed resistance. Epidemiologic cut-offs may thus be used to identify isolates that are less likely to respond to antimicrobial therapy because of acquired resistance mechanisms (Table 2). Surveillance data from the NICD (N.P. Govender, pers. comm., unpublished) obtained from *C. auris* bloodstream isolates from South African public and private-sector hospitals roughly align with tentative ECOFFs determined for 123 *C. auris* isolates.^22^ The US CDC has applied tentative non-validated clinical breakpoints developed for other *Candida* species to *C. auris* for epidemiological purposes; however, these may not necessarily be clinically relevant at an individual patient level.^27^ Susceptibility data for *C. auris* isolates published from multiple countries demonstrate uniformly high fluconazole MICs, with variable susceptibility to other azoles, echinocandins and amphotericin B.^10^ Some isolates may demonstrate high MICs to ≥ 2 antifungal classes (i.e. multidrug-resistant).

**TABLE 2:** Proposed cut-off values for *Candida auris* for 10 antifungal agents and corresponding South African surveillance MIC_90_ data.

Antifungal agent	Minimum inhibitory concentration (MIC) (µg/mL)		
	NICD surveillance data (MIC_90_)	Tentative ECOFF value	US CDC proposed cut-off value
Fluconazole†	256	≥ 128	≥ 32
Voriconazole‡	2	≥ 1	-
Itraconazole‡	0.25	≥ 0.25	-
Isavuconazole‡	-	≥ 0.5	-
Posaconazole‡	0.12	≥ 0.125	-
Caspofungin‡	-	-	-
Anidulafungin‡	0.25	≥ 0.25	≥ 4
Micafungin‡	0.12	≥ 0.25	≥ 4
Flucytosine‡	0.25	-	-
Amphotericin B‡	1	≥ 2	≥ 2

Note: MIC_90_, lowest concentration of the antifungal at which 90% of the isolates are inhibited. MIC_90_ data obtained from the National Institute for Communicable Diseases/Germs-SA surveillance for 344 bloodstream *C. auris* isolates. ECOFF, epidemiological cut-off value obtained via a derivatisation method using broth microdilution MICs obtained by the Clinical and Laboratory Standards Institute M27-A3 and European Committee on Antimicrobial Susceptibility Testing E, Def 7.3 methods. US CDC, US Centers for Disease Control and Prevention.

† , Resistant;

‡ , A high MIC has been obtained and the isolate has been referred to a reference laboratory.

This MIC indicates that use of this antifungal agent may be ineffective. Discuss with a clinical microbiologist or infectious diseases physician.

### Section 2: Surveillance and outbreaks

#### Recommendation 2.1: Should laboratory-confirmed cases of *Candida auris* infection and colonisation be routinely reported through surveillance?

1. There should be nationally coordinated surveillance for *C. auris* integrated into broader surveillance for antimicrobial resistance (AMR). The overarching goal is to prevent *C. auris* from becoming endemic in hospitals across South Africa.
2. At a facility level, all public-sector hospitals and private hospital groups should passively monitor the number of laboratory-confirmed cases of *C. auris* disease and colonisation.
3. At a national level, the NICD should conduct regular cross-sectional surveys in order to monitor epidemiological and geographical trends over time.

*Candida auris* is an emerging and multidrug-resistant pathogen that spreads rapidly in healthcare settings. The overarching goal of national surveillance is to provide information to prevent *C. auris* from becoming endemic in healthcare facilities and communities across South Africa and facilitate preparedness in laboratories for accurate detection and in IPC programmes for prevention and control.^30^ The objectives of surveillance should be:

- at a healthcare facility level:
  ■ to monitor the prevalence of culture-confirmed *C. auris* disease and colonisation
  ■ to detect outbreaks
- at a national level:
  ■ to detect emergence of antifungal resistance in strains of *C. auris* and thus guide empirical treatment
  ■ to describe potentially modifiable risk factors for invasive disease and death.

At a healthcare facility level, all public-sector hospitals and private hospital groups should passively monitor the number of cases of *C. auris* disease and colonisation by maintaining a line-list of culture-confirmed cases. The facility IPC practitioner(s) should be promptly notified of every *C. auris* case and should keep a record of the number of cases, by site of infection, wards where cases occurred and rates of infection, if possible, on a monthly basis. Facilities may be classified into three tiers (regular re-classification should be done by the facility IPC practitioners).

- Tier 1 (‘green status’): Facilities with no prior cases of *C. auris* disease or colonisation. Such facilities are requested to report their first cases to the NICD and/or the relevant district communicable disease control (CDC) team.
- Tier 2 (‘orange status’): Facilities with sporadic cases of *C. auris* infection or colonisation (i.e. < 12 cases in the past 6 months and/or < 3 units affected). Facilities are requested to report any increase in the number of cases compared to a baseline, units affected for the first time, or apparent clustering within a facility to the NICD and/or relevant district CDC team.
- Tier 3 (‘red status’): Facilities with relative endemicity (> 12 cases in the last 6 months and/or > 3 units with *C. auris* cases in the last 6 months) are requested to report any increase in the number of cases compared to a baseline or apparent clustering within a facility to the NICD and relevant district CDC team.

At a national level, NICD should conduct regular cross-sectional surveys as part of integrated AMR surveillance. These surveys could be scheduled at the same time every year and could be integrated with national point prevalence surveys for healthcare-associated infections (HAI) and AMR.^23^ NICD should coordinate nested epidemiologic studies through its existing surveillance platforms. *C. auris* is included in a list of alert organisms that South African healthcare facilities are encouraged to compile.^31^ Guidance has been issued from several other public health agencies across the world. US facilities are currently requested to report all cases to the CDC by using a dedicated email address.^32^ Public Health England (PHE) currently requests facilities to report all new cases of colonisation or infection to their local PHE Centre Health Protection Team. The European Centre for Disease Prevention and Control (ECDC) recommends that member states should consider laboratory-based notification of *C. auris* invasive disease and prospective data collection at national level. Surveillance systems for HAIs should be updated to include *C. auris* in the list of reportable pathogens associated with HAIs.

#### Recommendation 2.2: How should an outbreak of *Candida auris* be defined, reported and managed?

1. All suspected clusters/outbreaks should be reported to the relevant district CDC team and to the NICD in high-priority scenarios (refer to text below).
2. In a resource-constrained setting, outbreak response efforts should be focused on high-priority scenarios, as recommended in the text below.

An outbreak is defined as a sudden temporal increase in the number of cases of *C. auris* colonisation or infection within a unit or facility compared to a baseline, with epidemiological links which suggest clustering. The definition of an outbreak will not necessarily be the same for all units or facilities; therefore, each facility should be aware of their own tier status and distribution of prior cases within the facility. All suspected clusters or outbreaks should be reported by the facility IPC practitioner or laboratory to the relevant district CDC team and to the NICD in the following high-priority scenarios (Table 3). Not all outbreaks will require the same type of response. As resources for outbreak detection and response are limited, particularly in the public sector, urgent outbreak response efforts should be focused on:

- Clusters of cases in
  ■ patient groups who have not been previously described to be affected
  ■ units where the risk of horizontal transmission is high or consequences of disease are severe, for example neonatal or oncology units
  ■ facilities with no prior cases (i.e. Tier 1/green-status hospitals)
  ■ geographic regions with no or few prior cases
- Large outbreaks in facilities with or without relative endemicity (i.e. Tier 2 or 3 facilities).

**TABLE 3:** Suggested activities following detection of an outbreak of *Candida auris* in a healthcare facility.

Activity	Purpose
Notify relevant authorities	Obtain resources for prevention and control
Intensify infection prevention and control (IPC) measures, specifically contact precautions and environmental cleaning	Control outbreak, prevent further transmission
Isolate/cohort case patients	Limit transmission within a unit or facility
Contact screening	Inform further IPC measures, possibly limit transmission
Emphasise antifungal stewardship (AFS)	Possibly prevent further cases

Outbreak response activities may include, but are not limited to:

- Intensifying IPC measures (refer to Section 3), including screening of other high-risk patients, for example a patient who has been in a neighbouring bed to a case patient in an open ward and who is not known to have *C. auris* disease. Screening of facility personnel is not routinely recommended during an outbreak.
- Environmental screening, where appropriate.
- Emphasising AFS (Section 5).

Outbreak investigations reported from other countries describe response activities that have been effective. Following a large outbreak in a cardiothoracic facility in the United Kingdom, screening of all direct contacts was recommended. Screening of hospital personnel had a very low yield and was not recommended.^33^ In the United States, screening of close contacts of 77 case patients resulted in identification of an additional 45 patients with *C. auris* colonisation. Public health surveillance and ongoing investigations were recommended.^23^

### Section 3: Infection prevention and control

#### Recommendation 3.1: Which infection prevention and control precautions are necessary for patients colonised or infected with *Candida auris*?

Two sets of precautions are recommended (Table 4):

1. Standard precautions: These apply to all patients and in all situations and are designed to reduce the risk of transmission of microorganisms from both recognised and unrecognised sources of infection in healthcare settings.
2. Contact transmission-based precautions for patients known to be colonised or infected with *C. auris*: These are designed to interrupt transmission of epidemiologically important pathogens such as *C. auris* based on the contact route of transmission.

**TABLE 4:** Summary of recommendations for the prevention of transmission of *Candida auris*.

Measure	Description
Standard precautions	Strictly adhere to the five moments of hand hygiene^a^ including bare below the elbows and no jewellery (including rings, watches and bracelets). Wash hands when visibly soiled or after contact with blood and body fluids. Use a 70% alcohol-based hand rub on dry hands in all other instances. Monitor adherence to hand hygiene by visual inspection and auditing of adherence versus the number of opportunities.
Contact transmission-based precautions	Make gloves and disposable impervious aprons available. Wear disposable (impervious) gowns when there is close contact with a patient, for example turning a large patient where the healthcare worker’s uniform might be contaminated, or a high risk of blood and body fluid exposure. Wear eye protection and mask during procedures where there might be risks of splashes. Don all personal protective equipment (PPE) prior to entering the room and before touching a patient or the immediate environment (bed, linen, equipment, invasive devices and personal items). Remove and discard PPE and clean hands before leaving the patient’s room or, in semi-private room or multi-bed bay situation, before leaving the patient’s immediate vicinity. Visitors need not use PPE unless performing a nursing duty. Dedicate equipment to individual patients if possible, for example, blood pressure cuffs, thermometers. If equipment is shared, disinfect these according to the manufacturer’s guidelines between patient uses.
Isolation or cohorting	Accommodate each infected and/or colonised patient in a single room with en-suite facilities. Affix a ‘contact precautions’ sign to the door. If single rooms are not available, ‘cohort’ patients who are infected or colonised with the same pathogen (i.e. same species, similar susceptibility profile) in the same room. Ensure that the space between beds is adequate when patients are cohorted, that is, at least 2 m between the sides of the beds to allow adequate movement and use of mobile equipment without touching the other patient. Restrict the number of visitors at a single time.
Environmental cleaning	Clean rooms at least daily. Clean the room to reduce the bioburden and then disinfect with a sodium-hypochlorite solution (1000 parts per million). Clean and disinfect equipment (according to the manufacturer’s guidelines) after use if single-use items are not available. Handle all linen from infected or colonised patients as infectious linen, immediately place in a yellow plastic bag and wash separately at 65˚C for 10 min. All linen including bed curtains should be removed and laundered after discharge. Consider hydrogen peroxide fogging or wipes as an adjunctive measure when the patient vacates the room. There is insufficient evidence based on studies done in healthcare environments to currently recommend UV light disinfection.
Care bundles^b^	Adherence to the relevant care bundles should be monitored and measured. The following care bundles apply, where relevant: tracheostomy, central line-associated bloodstream infection (CLABSI), catheter-associated urinary tract infection (CAUTI), ventilator-associated pneumonia (VAP). All devices should be removed as soon as possible.
Patient movement	Notify receiving departments if patient is to be transported between departments. Notify the receiving hospital if the patient is transferred to another hospital or long-term care facility.
Training	Train cleaning personnel to correctly make sodium-hypochlorite solutions and how to clean. Educate patients, visitors and families on hand hygiene. Train multidisciplinary team members on IPC recommendations.

Note: The ‘five moments of hand hygiene’ is a phrase used by the World Health Organization to define the points at which hand hygiene should be performed in healthcare settings. These include the following ‘moments’: before patient contact, before an aseptic technique, after blood and body fluid exposure, after patient contact and after contact with the patient’s environment.^78^ A ‘care bundle’ is a structured way of improving the processes of care and patient outcomes. A care bundle is a group of evidence-based practices, which, when performed collectively and consistently, has proved to improve patient outcomes.

*Standard precautions* apply to all patients and in all situations, regardless of diagnosis or presumed infection/colonisation status. Standard precautions apply to blood, all other body fluids, secretions and excretions except sweat (regardless of whether they contain visible blood or not), non-intact skin and mucous membranes. As part of standard precautions, 70% alcohol-based hand rub is recommended for hand hygiene; a combination of chlorhexidine and alcohol may provide additional benefit.^34^ Personnel should perform hand hygiene before touching a patient, before a clean/aseptic procedure (e.g. inserting a peripheral line), after body fluid exposure, after touching a patient and after touching patient surroundings. Hand hygiene adherence should be measured with a standardised checklist and adherence should be monitored on a regular basis in all wards of a facility on a rotating basis. Routine hand sampling of staff to monitor adherence to hand hygiene is not recommended.

*Contact transmission-based precautions* (including isolation, cohorting and use of personal protective equipment such as disposable aprons and gloves) are not specific to *C. auris* and are recommended for several other multidrug-resistant organisms.^35^ Adherence to contact precautions should be monitored on a regular basis in all wards with patients who have contact precautions implemented because of *C. auris* infection and/or colonisation. If this level of monitoring is not possible, consider monitoring adherence primarily in the isolation unit where patients with *C. auris* are cohorted.

#### Recommendation 3.2: For how long should the infection prevention and control precautions remain in place for a patient with infection or colonisation?

1. Contact precautions should be implemented for the length of stay in an acute-care healthcare facility owing to prolonged colonisation, probable shedding of *C. auris* into the environment and no known effective methods for decolonisation.
2. Patients known to be colonised or infected with *C. auris* should ideally have contact precautions implemented when re-admitted to a healthcare facility.

The duration of colonisation is not clearly defined; in some cases, colonisation with *C. auris* may persist for many months, perhaps indefinitely.^3,36^ The optimal approach to reduce the skin or mucosal surface microbial load (i.e. decolonisation) of infected or colonised patients with *C. auris* has not been determined.^37^ While daily topical application of chlorhexidine gluconate 0.5% (including body washes and mouth gargles) has been recommended by at least one public health agency, patients have been documented to remain colonised with *C. auris* in prolonged outbreak settings despite this intervention.^33^ Similarly, the use of chlorhexidine-impregnated central vascular catheter dressings or topical nystatin has not been evaluated and these interventions are not recommended. Therefore, the most conservative approach for patients who are known to be infected or colonised with *C. auris* is to maintain contact precautions for the duration of admission. Patients known to be colonised or infected with *C. auris* should also be isolated when re-admitted to a healthcare facility; we have not specified a recommended time limit since the last admission because colonisation may be prolonged.

#### Recommendation 3.3: When is it appropriate to assess whether a patient or healthcare worker is colonised with *Candida auris* and how can colonisation status be ascertained?

1. Routine screening of all newly admitted patients for *C. auris* colonisation is not recommended.
2. Routine screening of healthcare personnel is not routinely recommended.
3. Screening might be considered in an outbreak situation to establish the prevalence of colonisation among epidemiologically linked patients, but not to establish colonisation of healthcare personnel.
4. Screening for colonisation can be performed by submitting skin swabs from the axilla and groin for selective culture (direct molecular tests are not currently available in South Africa).

Routine screening of all newly admitted patients is not feasible or recommended in a resource-constrained setting. However, screening may be considered in an outbreak situation to establish colonisation of epidemiologically linked patients. Epidemiologically linked contacts are defined as patients who are currently sharing a cubicle with a confirmed case. In areas that do not have cubicles, but are shared rooms with or without semi-permanent barriers, epidemiologically linked contacts include all patients in a shared physical area. Given the likely rapid colonisation potential of *C. auris*, the IPC practitioner could also consider screening any roommates the case patient may have had during the last month. Screening of healthcare personnel during an outbreak is not routinely recommended owing to the difficulty of evaluating the role of healthcare workers in the transmission of pathogens between patients and because the reported prevalence of carriage is relatively low.^33^

In an outbreak situation to establish colonisation of epidemiologically linked patients, specimens that could be submitted include the following: axillary skin swabs, groin skin swabs, nose/throat swabs, rectal swabs or stool samples, urine, wound fluid and respiratory tract specimens. The axillae and groin areas appear to be the most common and consistent sites of colonisation. We recommend that IPC practitioners should wait at least 48 h after administration of topical antiseptics, for example chlorhexidine, before collecting specimens for *C. auris* colonisation. An enrichment protocol has been described to optimise laboratory isolation of *C. auris* from colonisation samples.^14^ If a patient screens positive for *C. auris*, no further sampling is indicated. A negative colonisation screen should not be used as evidence to discontinue contact transmission-based precautions in a person with prior culture-confirmed invasive disease or colonisation; in such patients, it may be prudent to isolate but not cohort with other infected or colonised patients.

#### Recommendation 3.4: How should the immediate environment of patients infected or colonised with *Candida auris* be cleaned?

1. All surfaces should be cleaned daily with a neutral detergent and water and then wiped with a freshly constituted sodium-hypochlorite (1000 parts per million) solution. Other disinfectants such as quaternary ammonium compounds and ethyl alcohol are less effective and should not be used.
2. There is currently insufficient evidence to recommend routine ultraviolet (UV) light disinfection although hydrogen peroxide vapour or wipes may be considered.
3. Rooms/bathrooms or bed spaces should be terminally cleaned after the patient vacates the space.

Environmental surfaces are a reservoir for *C. auris.*^38^ Like *C. parapsilosis, C. auris* has been documented to persist on plastic surfaces for up to 28 days in a controlled environment mimicking a healthcare setting.^14^ *C. auris* forms biofilms which may enhance its persistence in the environment.^11,12,13^ Guidance for environmental cleaning is not consistent, with variability across the recommendations from several public health agencies.^37^

*Daily cleaning*: All surfaces and equipment should be cleaned daily with a neutral detergent and water. Standard cleaning should be followed by wiping surfaces with an appropriate disinfectant. Chlorine-based disinfectants effectively kill *C. auris* in suspension and inoculated on surfaces.^16,17,34,39^ Chlorine disinfectants also kill other multidrug-resistant pathogens such as methicillin-resistant *Staphylococcus aureus* and carbapenem-resistant *Enterobacterales*. A sodium-hypochlorite solution (1000 parts per million) is recommended for daily cleaning. While some public health agencies recommend higher concentrations of sodium hypochlorite, there is limited evidence to support this and the guideline development group had concerns about corrosive damage to re-useable equipment and adverse (noxious) effects on personnel working with a concentrated solution.^37^ New chlorine-based solution should be prepared daily at a minimum and stored away from sunlight and heat to preserve potency. Cleaners should be given clear instructions how to prepare the chlorine solutions, including pictorial depictions of the dilution process. Cleaning should proceed from cleanest to dirtiest areas, for example cleaning patient’s bedside table prior to cleaning the commode. Cleaning supplies, for example mop heads and buckets, should be decontaminated regularly. Adequate contact time should be allowed with the disinfectant (at least 3 min).^16^ Frequently touched areas should be cleaned and disinfected more often (at least twice a day). Quaternary ammonium compounds and ethyl alcohol appear to be less effective for environmental disinfection of *C. auris* and should not be used.^17,37,39^ Routine environmental sampling to culture *C. auris* from patient care areas as a proxy for efficacy of terminal cleaning is not recommended.

*Equipment*: Single-use equipment is preferred, but if it is not available, dedicated equipment should be used for the duration of the patient’s stay. Equipment should be cleaned thoroughly and disinfected according to the manufacturer’s recommendations. Surfaces of equipment should be cleaned adequately to remove dirt and organic material prior to disinfection; sodium hypochlorite is less effective in the presence of organic material.

*Terminal cleaning*: Terminal cleaning protocols must be strictly adhered to using checklists which are completed by the IPC team. Terminal cleaning should involve cleaning and disinfection of all items and surfaces in the patient care area or room as well as laundering or changing any difficult-to-clean items, for example curtains and movable partitions. Terminal cleaning or disinfection should begin with removing all disposable items (e.g. suction canisters, glove boxes, tubing and waste) and items intended to be removed and cleaned outside patient care area (e.g. laundry items). All surfaces and equipment should be cleaned with a neutral detergent and water and then wiped with a sodium-hypochlorite solution. Although higher concentrations of this solution have been used for terminal disinfection in outbreaks,^33^ we recommend 1000 parts per million. Hydrogen peroxide vapour or wipes appear to be effective against *C. auris* and may be added as an additional measure after cleaning and disinfection.^16,17,39^ There is limited evidence for the use of UV light disinfection for *C. auris*. A recent study examining the efficacy of UV-C light (254 nm) showed that an exposure time of 20 min was required to destroy *C. auris*; this was substantially longer than the time required to kill MRSA.^40^ It is important to note that ‘non-touch’ environmental disinfection methods, such as hydrogen peroxide vapour and UV light, cannot replace traditional methods and may only be considered an adjunct to traditional cleaning and contact disinfection of the environment.

### Section 4: Treatment of invasive and non-invasive *Candida auris* disease

#### Recommendation 4.1: What are the suggested treatment regimens for confirmed or strongly suspected invasive *Candida auris* disease in adults and children?

1. In the vast majority of adults, an echinocandin is recommended as first-line treatment. Amphotericin B deoxycholate is an alternative agent in settings where echinocandins are unavailable and is recommended for central nervous system, urinary tract or eye infections.
2. Among children aged < 2 months, the initial treatment of choice is amphotericin B deoxycholate 1 mg/kg daily,
3. Among children aged > 2 months, an echinocandin is recommended for the initial treatment.

Early aggressive treatment of invasive *Candida* disease is vital for improved outcomes in critically ill adults.^41^ In the vast majority of adults with invasive *Candida* disease (including *C. auris*), an echinocandin is recommended as first-line treatment.^42^ Amphotericin B deoxycholate is an alternative agent in settings where echinocandins are unavailable. Amphotericin B is also preferred in invasive infections of the central nervous system, eye and urinary tract.^43^ Although amphotericin B deoxycholate is known to exhibit concentration-dependent killing activity, continuous infusion may be associated with better tolerability and less renal toxicity and may therefore be desirable in those settings where this is possible.^44^ Azole antifungal agents such as fluconazole and voriconazole are not recommended as initial treatment for suspected or confirmed *C. auris* invasive disease. In many centres, reduced susceptibility or high-level resistance has been demonstrated to these agents.^10^ While posaconazole MICs for South African *C. auris* strains are relatively low (MIC_50_ of 0.12 mg/L), the first-line use of this agent should only be considered in consultation with an ID specialist or a specialist with a particular interest in this field. Posaconazole is currently only available as an oral formulation in South Africa. Clinicians are advised to check for potential drug–drug interactions and adverse effects when prescribing antifungals. A useful antifungal interactions smartphone application can be accessed at https://www.aspergillus.org.uk/content/antifungal-drug-interactions. Currently available antifungal agents with efficacy against *C. auris* are shown in Table 5.

**TABLE 5:** Antifungal agents for adults with invasive disease.

Agent	Dose	Dose adjustments with renal dysfunction	Common adverse effects
Caspofungin Micafungin Anidulafungin	Loading dose 70 mg IV, then 50 mg; IV daily 100 mg IV daily; Loading dose 200 mg IV, then 100 mg; IV daily	Dose as in normal renal function	Fever, thrombophlebitis, headache, raised serum transaminases
Amphotericin B deoxycholate Liposomal amphotericin B	1 mg/kg IV daily; 5 mg/kg IV daily	Avoid deoxycholate formulation if baseline CrCl < 50 mL/min. If baseline CrCl ≥ 50 mL/min, deoxycholate can be used but must ensure adequate hydration and avoid using other nephrotoxic agents	Deoxycholate > lipid formulations: nephrotoxicity, hypokalaemia, hypomagnesaemia, fever, pain at injection site
Flucytosine†	25 mg/kg 6 hourly PO (total daily dose: 100 mg/kg)	If CrCl reduces to below 40 mL/min, give the same 25 mg/kg dose but increase the interval between doses: 20 mL/min–40 mL/min, 12 hourly; 10 mL/min–20 mL/min, every 24 h; < 10 mL/min, > 24 h	Photosensitivity, gastrointestinal toxicity, hepatotoxicity, haematological toxicity
Posaconazole‡	400 mg BD PO with meals	Dose as in normal renal function	Gastrointestinal toxicity, raised serum transaminases, skin rash, hypokalaemia

IV, intravenous infusion; bd, twice daily; PO, per os; CrCl, creatinine clearance = (140 – age) * (weight in kg)/(72 * serum creatinine in mg/dL) [Multiply result by 0.85 for women].

† , 5-FC is available through Section 21 application through the South African Health Products Regulatory Authority (SAHPRA), formerly the SA Medicines Control Council. 5-FC should not be used as monotherapy but always in combination with another antifungal agent. The laboratory should determine 5-FC minimum inhibitory concentrations if this agent is being considered for use;

‡ , *C. auris* is usually not susceptible to fluconazole and voriconazole.

*Neonates or infants aged < 2 months*: For neonates or infants less than 2 months old, amphotericin B deoxycholate should be used as a first-line treatment of invasive infections (Table 6).^45^ Amphotericin B is efficacious and well tolerated in neonates. Fluconazole should not be used for treatment of *C. auris*; fluconazole also has no activity against azole-resistant strains of *C. parapsilosis* which are endemic in some South African neonatal units.^18^ Echinocandin use should be limited and reserved for cases of salvage therapy or where severe toxicity precludes the use of amphotericin B. There is no evidence for combination antifungal therapy in this age group for the treatment of *C. auris*.

**TABLE 6:** Antifungal agents for children younger than 2 months of age with invasive disease.

Agent	Dose
Amphotericin B deoxycholate	1 mg/kg IV daily
Caspofungin	25 mg/m^2^ IV daily
Micafungin	10 mg/kg IV daily

*Children aged > 2 months*: Echinocandins are the preferred agents for most cases of candidaemia and invasive candidiasis (Table 7). Exceptions include infections of the central nervous system, eye and urinary tract where amphotericin B deoxycholate should be used. Patients should be closely monitored for treatment failure, as indicated by persistently positive clinical cultures. Switching to amphotericin B should be considered if the patient has persistent fungaemia for > 5 days or is unresponsive to echinocandin treatment. Fluconazole should not be used for treatment of *C. auris*. No supporting evidence exists for combination antifungal therapy in children.

**TABLE 7:** Antifungal agents for children younger than or equal to 2 months of age with invasive disease.

Agent	Dose
Caspofungin	Loading dose: 70 mg/m^2^ IV daily, then 50 mg/m^2^ IV daily
Micafungin	2 mg/kg IV daily, with option to increase to 4 mg/kg IV daily in children > 40 kg
Anidulafungin	Not approved for use in children
Amphotericin B deoxycholate	1 mg/kg IV daily

#### Recommendation 4.2: How should the source of infection be identified and controlled in adults and children?

*Candida auris* bloodstream infections are usually associated with healthcare settings and occur among patients with intravascular catheters and prosthetic devices. While many of these bloodstream infections represent candidaemia alone, attempts to exclude deep-seated infections, such as infective endocarditis, osteomyelitis, meningitis, pyelonephritis and endophthalmitis (by dilated retinal examination), should be undertaken.^23,46^ This will influence treatment duration and penetration of antifungal agents into the source area will need to be considered. In such cases, consultation with an ID specialist (or a specialist with a particular interest in this condition) is recommended. *C. auris* fungaemia may be difficult to control. Without adequate and appropriate source control, antifungal treatment alone may be futile. All attempts should be made to remove or replace indwelling central venous and arterial devices, as well as urinary catheters. Infected prosthetic material such as heart valves, shunts and bone fixation devices should be surgically removed, where feasible. Any collections should be drained. In addition, risk factors for candidaemia should be modified where possible. A summary of recommended source control and risk factor modification measures is presented in Table 8. In neonates with blood and/or urine cultures positive for *C. auris*, a lumbar puncture and a dilated retinal examination are recommended. If cultures are persistently positive, imaging of the genitourinary tract, heart, liver and spleen should be performed. Central venous catheter removal is strongly recommended. Surgical intervention should be considered for fungal balls in the kidneys and for endocarditis.^42^

**TABLE 8:** Source control and risk factor modification measures.

Source/risk factor	Suggested intervention
Indwelling venous/arterial catheters	Remove or replace
Urinary catheter	Remove or replace
Infected prosthetic material	Remove or replace
Collections/abscesses	Drain surgically or insert pigtail
Antibiotics	Stop/de-escalate/use only if deemed absolutely necessary
Corticosteroids	Stop/wean
Immunosuppressants	Stop/wean/modify
Total parenteral nutrition	Change to enteral nutrition, if possible

#### Recommendation 4.3: How should response to treatment be monitored following a confirmed episode of invasive disease?

Blood cultures and laboratory or biochemical markers (including peripheral white cell count [WCC], platelet count and C-reactive protein [CRP]) should be repeated at least three times a week to monitor clearance after candidaemia is confirmed by blood culture.

Blood cultures for initial diagnosis of candidaemia or monitoring clearance of bloodstream infection should be collected using strict aseptic technique. Among adults, each blood culture bottle should be inoculated with at least 10 mL of blood from a peripheral venepuncture site (total volume of a blood culture set: up to 40 mL–60 mL).^47^ Follow-up blood cultures can help to determine the appropriate duration of antifungal therapy. Blood cultures should be repeated at least three times a week in order to document clearance of candidaemia.^42^ Many laboratories routinely perform MIC testing on all invasive *Candida* strains: MICs of subsequently cultured strains should be closely monitored to identify antifungal resistance which may require treatment modification.^23^ In addition, we suggest that markers such as a peripheral WCC, platelet count and CRP should be measured regularly to assist with treatment monitoring and clinical response. Kidney function and electrolytes (especially potassium and magnesium) should be monitored closely, particularly if the patient is being treated with amphotericin B deoxycholate.^48^ Serum procalcitonin levels usually remain between 2.0 ng/mL and 2.5 ng/mL among patients with invasive *Candida* infections; thus, procalcitonin is not a useful marker for monitoring response to treatment.^49^ A negative serum (1,3) beta-D-glucan (BDG) level may be a useful adjunct to exclude a diagnosis of candidaemia in critically ill adults.^42,50,51^ There are no published data on the utility of serum BDG for initial diagnosis of invasive *C. auris* infection. A decrease in serially collected serum BDG levels during treatment for candidaemia is associated with clinical/microbiological resolution.^52,53^ However, no recommendation can be made on the use of serum BDG for monitoring response to *C. auris* infection because no data are currently available.

#### Recommendation 4.4: What is the recommended duration of treatment for an episode of invasive disease?

If no evidence of a deep-seated fungal infection is found (e.g. infective endocarditis, meningitis, osteomyelitis, pyelonephritis, endophthalmitis or prosthetic infection) and disease is thus considered uncomplicated, antifungals are recommended to be continued for a minimum period of 2 weeks from the date of clearance of the candidaemia, as documented by negative blood cultures, in conjunction with clinical resolution.^42^ Treatment of deep-seated or complicated infections is usually prolonged and should be in consultation with an ID specialist.

#### Recommendation 4.5: When may combination antifungal treatment be considered for invasive disease?

1. Combination therapy is not recommended among clinically stable patients with invasive *C. auris* disease. There is no evidence for combination antifungal therapy in children for the treatment of *C. auris*.
2. Among a minority of critically ill patients with septic shock, initial combination therapy with an echinocandin plus either amphotericin B or flucytosine may be considered for a short period until antifungal susceptibility results are available.
3. In addition, combination therapy may be considered, following consultation with an ID specialist, in patients with persistent fungaemia, relapsing fungaemia and recurrent fungaemia where source control has been addressed.
4. For infective endocarditis and meningitis, flucytosine (if available and the isolate is susceptible) may be added to the treatment regimen.
5. Combination therapy in the absence of adequate source control is futile.

Although there is currently no evidence for combination therapy in any patient population with invasive *C. auris* disease, crude (unadjusted) mortality is unacceptably high,^54^ especially among critically ill and immunosuppressed patients. We therefore recommend initial combination therapy in the sub-groups mentioned above, along with prompt source control. Where initial combination antifungal therapy is commenced among patients in septic shock (defined as a mean arterial blood pressure [MABP] ≤ 65 mmHg or requiring vasopressor support and lactate > 2 mmol/L^55^), daily evaluation for the ongoing requirement of combination therapy should be reviewed while awaiting antifungal susceptibility results and/or clinical stabilisation of the patient. Following susceptibility testing results, de-escalation to a single antifungal agent to which the pathogen is susceptible should be considered, provided that the patient has clinical and laboratory improvement and has undergone adequate, appropriate source control measures. This should happen within a 72-h time frame. Combination therapy may be considered among patients who remain blood culture positive after 5–7 days (defined as persistent fungaemia) despite attempts at suitable source control, appropriate antifungal dosing and optimised antifungal penetration to the site of infection; isolate MICs should be reviewed by a clinical microbiologist. Patients who become culture positive following completion of initial antifungal treatment and presumed clearance of infection (defined as recurrent fungaemia), as well as patients who become culture positive after a period of negative cultures while still receiving appropriate treatment (defined as relapsing fungaemia), may also be considered for combination therapy, as well as detailed further investigations. In all patients, appropriate antifungal dosing and source control are of paramount importance. Treatment of these complex patients is recommended to be continued in consultation with an ID specialist and clinical microbiologist.

#### Recommendation 4.6: How should a patient be managed if *Candida auris* is isolated from a non-sterile body site?

Isolation of *C. auris* from a non-sterile body site (such as skin, rectum, upper or lower respiratory tract or urinary tract) in the absence of markers of inflammation or organ dysfunction and clinical signs of infection is usually an indication of colonisation and not disease. In this setting, antifungal treatment should be avoided; however, colonisation may prompt removal of indwelling devices (such as urinary catheters) and institution of appropriate IPC measures (refer to Section 3). In the presence of clinical signs of infection, attempts to isolate *C. auris* from a sterile site (such as blood, CSF, tissue, central venous catheters, etc.) should be made. Ancillary markers of fungaemia such as a serum BDG assay may be useful to exclude cases of candidaemia (this assay has excellent negative predictive value [NPV] among critically ill adults).^42,50^

### Section 5: Antifungal stewardship

#### Recommendation 5.1: When is antifungal prophylaxis indicated for critically ill patients and which agent should be used?

1. Prophylaxis should be considered for the following high-risk patient groups:
  - Surgical patients:
    - presenting with anastomotic leakage after abdominal surgery
    - re-operation of the digestive tract during the same hospitalisation
  - Neonates:
    - extremely low birth weight (ELBW) infants (body weight [BW] < 1000 g) in neonatal units with a baseline rate of invasive candidiasis of 5% – 10%
2. Depending on local epidemiology and patient population, fluconazole, echinocandin or amphotericin B may be considered. Fluconazole prophylaxis should be avoided in settings with *C. auris* or azole-resistant *C. parapsilosis*.
3. The optimal duration of prophylaxis is not known.

Antifungal prophylaxis among non-neutropenic critically ill patients remains controversial, including among surgical patients with severe acute pancreatitis.^56,57^ While fluconazole prophylaxis may reduce the incidence of invasive candidiasis in critically ill adults and neonates, emergence of resistance in *Candida* species other than *C. albicans* is a concern with universal prophylaxis in this high-risk population. Previous exposure to antifungals is associated with a shift in *Candida* species distribution and an upward antifungal MIC ‘creep’.^58^ In addition, the threat of emergence of cross-resistance to both triazoles and echinocandins exists, as described in *Candida glabrata*, a species which notoriously sequentially acquires and expresses multiple resistance genes.^59^ The dominance of triazole-resistant *C. parapsilosis* causing bloodstream infections in South Africa was recently confirmed, particularly in ICU patients in the private sector.^18^ Overuse of triazoles for prophylaxis and treatment of candidaemia and other fungal infections may have led to the emergence and subsequent nosocomial transmission of these triazole-resistant strains. Similar factors may apply to *C. auris* in South Africa.^9^ The epidemiology of candidaemia in South Africa is unusual: *C. albicans* and *C. parapsilosis* dominate in the public and private sectors, respectively.^18^ Multidisciplinary AFS teams should choose prophylactic agents based on local surveillance data. The recommended antifungal options and doses for prophylaxis in adults and children are summarised in Table 9.^42,60^ However, the optimal duration of prophylactic treatment is not known.^61^

**TABLE 9:** Recommended antifungal agents and doses for prophylaxis among adults and children.

Patient group	Antifungal agent	Loading dose	Daily maintenance dose
Adults	Fluconazole	800 mg (12 mg/kg)	400 mg (6 mg/kg)
	Amphotericin B	-	0.5 mg/kg–1 mg/kg
	Caspofungin	70 mg	50 mg
	Micafungin	-	100 mg
	Anidulafungin	200 mg	100 mg
Neonates	Fluconazole	-	-
	GA< 30 weeks or < 1000 g	-	3 mg/kg – 6 mg/kg/dose twice a week
	GA 30–40 weeks	-	6 mg/kg/dose 48 hourly
Infants and children > 1 month	Fluconazole	-	6 mg/kg/day
	Amphotericin B†	-	1 mg/kg/24 h D1-7 1 mg/kg/48 h after D7

GA, gestational age.

† , Amphotericin B is recommended only in very rare instances;

#### Recommendation 5.2: How can patients be identified for early antifungal treatment?

There is insufficient evidence to make a firm recommendation on the optimal strategy to identify patients who may benefit from early antifungal treatment.

From a clinical point of view, early diagnosis and treatment of invasive candidiasis is the key to reduction in mortality. To minimise the negative impact of this infection, several management strategies had previously been described: antifungal prophylaxis, empirical therapy, pre-emptive therapy and directed culture-based treatment. However, both universal antifungal prophylaxis and empirical therapy (based on the persistence of fever non-responsive to antibacterial agents and a combination of risk factors) may overexpose the patients to antifungal treatment, potentially increasing antifungal resistance.^62^ Notably, up to 70% of critically ill patients receive systemic antifungal therapy although they have no documented invasive fungal infection,^63^ suggesting a need for alternative strategies. With the use of biomarkers such as the serum BDG assay and to simplify auditing of AFS process measures, the concepts of pre-emptive or empirical therapy should be substituted by ‘early’ antifungal treatment. Identifying patients at risk for invasive candidiasis includes recognition of a combination of risk factors. The *Candida* score was developed for critically ill non-neutropenic adults in Spanish intensive care units (ICUs) and is calculated by adding the following scores for each risk factor that is present: 1 (total parenteral nutrition), 1 (surgery), 1 (multifocal *Candida* species colonisation) and 2 (severe sepsis).^64^ Such predictive scores can help distinguish *Candida* colonisation and invasive candidiasis in ICUs, permit selection of high-risk patients who may benefit from early antifungal therapy and can also be used by AFS teams.^65^ However, given the low positive predictive values (PPVs) of such scores, many prescribed antifungal regimens have been shown to be unnecessary.^66^ In contrast, predictive scores have far better NPVs.^67^

Studies using non-culture-based assays, particularly serum BDG, together with a *Candida* score, have aided in establishing whether initiation of antifungal therapy in at-risk patients followed by close follow-up and discontinuation of antifungal therapy when invasive candidiasis is excluded has an impact on the outcomes of ICU patients. Combining BDG and the *Candida* score improves the sensitivity and NPV compared with either serum BDG or the *Candida* score alone.^63^ Using this approach, antifungal therapy was safely avoided in 73% of treatment-eligible ICU patients and treatment duration was shortened in another 20% of patients.^68^ In another cohort, early discontinuation of antifungal therapy (initiated in high-risk ICU patients following a positive *Candida* score ≥ 3) based on two consecutive negative serum BDG tests appeared to be a reasonable AFS strategy such that the combined assay is potentially usable and safe for the therapeutic decision-making process and discontinuing of early antifungal therapy.^69^ Similar outcomes were observed in a biomarker-based strategy using an algorithm involving serum BDG, mannan and anti-mannan assays.^70^ A recent study also aimed to assess the combined performance of serum BDG and procalcitonin to differentiate between invasive candidiasis and bacteraemia.^71^ When both markers indicated invasive candidiasis (BDG ≥ 80 pg/mL and procalcitonin < 2 ng/mL), they had a higher PPV (96%) compared to 79% and 66% for BDG or procalcitonin alone, respectively. When both markers indicated bacteraemia (BDG < 80 pg/mL and procalcitonin ≥ 2 ng/mL), the NPV for invasive candidiasis was similar to that of BDG used alone (95% vs. 93%). The combined use of procalcitonin (PCT) and β-D-glucan (BDG) could therefore be helpful in the diagnostic workflow for critically ill patients with suspected candidaemia. The data suggest that the concurrent use of the *Candida* score, BDG and other biomarkers may improve diagnostic stewardship in ICU patients at risk for *Candida* sepsis, but additional investigations are needed and their use as AFS tools remains to be established. In addition, the negative BDG cut-off < 80 pg/mL for *C. auris* and *Candida* species other than *C. albicans* in South Africa needs to be confirmed.

#### Recommendation 5.3: Which antifungal stewardship interventions should be considered in acute healthcare settings and how should these be implemented?

1. Implementation of AFS is recommended for all South African acute-care hospitals.
2. Multidisciplinary teams involving the necessary expertise should develop, implement and monitor AFS interventions.
3. Prospective audit and feedback is the recommended choice for the approach to AFS in South Africa, although other options may be considered in settings with limited resources. Targeted antifungal process measures should be audited as an AFS bundle.
4. AFS programmes are safe, irrespective of whether restrictive, structural and persuasive interventions are implemented alone or in combination.

No specific AFS programmes focusing on *C. auris* have yet been designed, but it is likely that an environment with high and inappropriate antifungal utilisation will favour the emergence of multidrug-resistant fungi. Changes in the distribution of *Candida* species may have impact on treatment recommendations because of differences in susceptibility to antifungal agents among species, but previous exposure to antifungal agents has likely contributed to this shift in species distribution.^62^ Inappropriate use, as opposed to overuse, also needs to be considered. This was highlighted in a bedside audit of antifungal use in patients admitted to a general hospital, where 57% of the prescriptions were found to be sub-optimal.^72^ Reasons for inappropriate use included inappropriate choice, dosing, de-escalation and duration of treatment. While an overall reduction in antifungal consumption is necessary, using the correct agent at the correct dose for the correct duration is also important. In support of this, a 3-year comprehensive AFS programme not only resulted in improved overall utilisation but also a significant decrease in fluconazole consumption (from 242 to 117 defined daily doses [DDDs] per 1000 patient-days) which was associated with a significant reduction in the incidence of *C. glabrata* and *C. krusei*.^61,73^ Therefore, to reduce overall consumption, enhance appropriate use of antifungal therapy and improve patient outcomes while minimising the risk of emergence of resistance, the implementation of an AFS programme is recommended in all South African hospitals.

Multidisciplinary teams encompassing the necessary expertise (in pharmacy, clinical microbiology, infectious diseases, internal medicine, surgery, paediatrics and anaesthetics) are an international recommendation for AFS.^74,75^ Given the lack of ID human resources in most South African hospitals, utilising existing multidisciplinary resources in a collaborative manner may enable an AFS programme to be embedded in routine practice.

Depending on resources, circumstances and the health sector in South Africa, restrictive stewardship interventions (such as formulary restriction, prior authorisation, therapeutic substitutions and automatic stop orders), structural interventions (such as changing from paper to computerised records, rapid laboratory testing, therapeutic drug monitoring, computerised decision support systems and the introduction of quality monitoring mechanisms), persuasive strategies (such as distribution of educational materials, educational meetings and outreach visits, local consensus processes, reminders provided verbally, on paper or on computer) and prospective audit, intervention and feedback should be considered.^76^ However, prospective audit, intervention and feedback has been shown to be a very effective and safe antibiotic stewardship strategy in South African hospitals, particularly in settings without ID specialists.^74^ Potential multi-component AFS process and outcome measures for clinician, pharmacist and/or ICU nurse audits are proposed in Table 10.

**TABLE 10:** Multi-component antifungal stewardship targets and corresponding recommended process or outcome measures.

Target	Recommended process measures	Recommended outcome measures (per unit)
Accountable justification	Did the clinician provide free-text justification for prescribing an antifungal agent (i.e. prophylaxis vs. ‘early’ AF therapy)? If for prophylaxis, was the antifungal agent prescribed according to consensus evidence-based indications?	-
Diagnostic stewardship	Was ‘early’ antifungal therapy based on risk factors? If based on risk factors, was a predictive score calculated? Were blood specimens for BDG and PCT levels obtained? Were blood cultures submitted?	-
‘Early’ initial antifungal choice and dose	Was the chosen antifungal agent consistent with guidelines? Was the dose prescribed compliant with guidelines? Where applicable, was a loading dose prescribed? Was the dose adjusted according to body weight, liver and renal function?	-
Time from prescription to administration (‘hang-time’)	Was the antifungal agent administered within 1 h?	-
Post-prescription review (48–72 h)	Was antifungal therapy discontinued in patients pending clinical condition and biomarker results (e.g. serum BDG, PCT)? If blood cultures became positive, was antifungal therapy de-escalated to a narrow-spectrum agent, pending susceptibility results?	-
Source control	In case of a positive blood culture, were existing CVCs removed within 24 h of diagnosis?	-
Duration of therapy for sepsis	Was an antifungal agent prescribed for a total duration of 14 days after first negative blood culture?	-
Length of stay	-	ICU stay Candidaemia-related stay
Mortality	-	30-day crude mortality Candidaemia-related mortality
Longitudinal ecological impact	-	Antifungal susceptibility profile Species distribution
Antifungal consumption	-	Overall antifungal consumption Echinocandin consumption Triazole consumption Amphotericin B consumption

BDG, (1,3)-β-D-glucan; PCT, procalcitonin; CVC, central venous catheter; ICU, intensive care unit: MDR, multi-drug resistant.

Antifungal stewardship process measures (Table 10) should preferably be audited as an ‘AFS bundle’, which is defined as a small set of evidence-based interventions for a defined patient population and care setting. In contrast to check lists, adherence to bundle components is measured using an all-or-nothing measurement, with a goal of ≥ 95%. As mentioned, the first step in the development and implementation of AFS is to build a multidisciplinary team.^74,75^ Using AFS bundles and all-or-nothing measurement may change the way care is provided for at-risk patients in important ways because bundles not only facilitate but also promote awareness that the entire care team must work together in a system designed for reliability.

The beneficial impact of ‘bundles’ on clinical outcomes in patients with invasive candidiasis was confirmed for the first time recently.^77^ The composite adherence to nine measures (all-or-nothing) was only 6.9% in a Japanese study, but there was a significant difference in clinical success between patients with and without adherence (92.9% vs. 75.8%). When step-down oral therapy was excluded from the measures, adherence to the bundles was shown to be an independent predictor of clinical success (odds ratio [OR] 4.42, 95% confidence interval [CI] 2.05–9.52) and mortality (OR 0.27, 95% CI 0.13–0.57). Notably in none of the studies in Appendix 1, Table 1-A1, where the impact of various AFS interventions for invasive candidiasis in a variety of settings including non-academic hospitals has been summarised, were patient outcome measures negatively affected. This included length of stay, re-admissions, length of hospitalisation, time until clearance of candidaemia, persistent candidaemia, recurrent candidaemia, triazole-resistant *Candida* species other than *C. albicans* and mortality compared to the pre-implementation phase.

## Appendix 1

**TABLE 1-A1:** Impact of antifungal stewardship programmes on non-patient–related outcome measures.

Reference	Study design and duration	Strategy: Restrictive (R), persuasive (P), structural (S)	Outcome measures				
			Overall AF reduction†		AF cost reduction	
			%	*p*	%	Saving
Cook et al.^79^	Pre-post quasi-experimental, 4 years	Formulary restrictions (R) Post-prescription review and feedback (*n* = 2 measures) (P)	28	0.02	20	-
Swoboda et al.^80^	Pre-post quasi-experimental, 3 years	Institutional practice guidelines (P) Post-prescription review (P)	ND	ND	50	€298 304 (pre-post)
Apisarnthanarak et al.^73^‡	Pre-post quasi-experimental, 3 years	Formulary restrictions (R) Post-prescription review and feedback (*n* = 5 measures) (P) Institutional treatment guidelines (P) Dedicated AF prescription chart and AFS ward rounds (P) Scheduled educational programmes (P) Dose-adjustment tool (S)	59	< 0.001	-	US$ 31 615 (pre-post)
Standiford et al.^81^	Three-phase interventional, 7 years	Preauthorisation (R) Post-prescription review and feedback (*n* = 4 measures) (P) Institutional treatment guidelines (P) Computer decision support (S)	ND	ND	45.8	US$130 000 (pre-post)
Lopez-Medrano et al.^82^	Pre-post non-randomised, 1 year	Post-prescription review and feedback (*n* = 4 measures) (P)	V -31.4; C -20.2	-	11.8	US$370 680 (pre-post)
Antworth et al.^83^§	Pre-post quasi-experimental, 6 months	Post-prescription review and feedback (*n* = 6 measures) (P) (Bundle) -	ND	ND	ND	ND
Guarascio et al.^84^	Matched-controlled, 6 months	Post-prescription bundle review and feedback (*n* = 4 measures) (P) Caspofungin only	50 (DOT)	0.001	-	US$1013 (per patient)
Mondain et al.^85^¶	Prospective observational, 6 years	Post-prescription review and feedback (*n* = 4 measures) (P) Institutional treatment guidelines (P) Scheduled educational programmes (P) AF order forms (S) TDM voriconazole and posaconazole (S) Diagnostic tools for IC (S)	38	-	56	€682 409
Alfandari et al.^86^	Retrospective observational, 9 years	Post-prescription ID consultation (P) Institutional treatment guidelines (P) Scheduled educational programmes (P) AF order forms (S)	40	-	ND	ND
Micallef et al.^87^	Prospective observational, 1 year	Post-prescription review and feedback (*n* = 4 measures) (P) High cost AFs only TDM voriconazole (S)	ND	ND	-	£178 708 (annum)
Takesue et al.^77^	Cluster non-randomised, 1 year	Post-prescription review and feedback (*n* = 9 measures) (P) (Bundle)	ND	ND	ND	ND

AF, antifungal; y, year; m, month: ND, not determined; V, voriconazole; C, caspofungin; DOT, days of therapy.

† , Unless otherwise stated, overall consumption was expressed as defined daily doses/1000 patient-days;

‡ , A significant reduction in inappropriate antifungal drug use was documented from 71% during the pre-intervention period to 24% during the post- intervention period (*p* < 0.001);

§ , A significant increase in composite compliance to all bundle measures in the AFSP group versus the control group was demonstrated (78.0% vs. 40.5%, *p* = 0.0016);

¶ , Improved compliance was achieved for the timing of antifungal treatment (*p* = 0.0025), recommended first-line therapy (*p* = 0.0025), duration of therapy (*p* = 0.46) and the removal of central venous catheters (*p* = 0.27), compared with pre-AFS implementation.
